# Draft genome of a polyurethane-utilizing bacterium *Pseudomonas* sp. PHC1

**DOI:** 10.1128/mra.00027-25

**Published:** 2025-06-12

**Authors:** Jooho Chung, Arum Han, Kung-Hui Chu

**Affiliations:** 1 Zachry Department of Civil and Environmental Engineering, Texas A&M University 14736, College Station, Texas, USA; 2 Department of Electrical and Computer Engineering, Texas A&M University 14736, College Station, Texas, USA; California State University San Marcos, San Marcos, California, USA

**Keywords:** polyurethane, biodegradation, *Pseudomonas*, plastic pollution

## Abstract

We report a draft genome sequence of *Pseudomonas* sp. PHC1, capable of utilizing polyurethane (PUR) as sole carbon and sole nitrogen sources.

## ANNOUNCEMENT

Polyurethanes (PUR), representing 8% of total plastic production, are the sixth most widely produced plastics globally. Approximately 70% of the total PUR produced contributes to marine plastic pollution, and PUR is rapidly ingested and bioaccumulated by marine organisms, leading to ecosystem disruption and potential human health risks (1, 2). Microbial degradation of PUR, facilitated by enzymes such as esterases and ureases, offers a promising solution for mitigating this pollution (3). We previously isolated a PUR-utilizing bacterium, *Pseudomonas* sp. strain PHC1 (hereafter referred to as strain PHC1), from estuary sediments of Galveston Bay, Texas (latitude of 29.676319, longitude of −94.781676) (4). The isolation process is briefly described below. Biodegradation of a plasma-treated PUR foam embedded in the estuary sediments in an aerobic microcosm for six months was observed according to SEM images and Fourier Transform Infrared spectroscopy (FTIR) analysis of the post-incubated PUR surface. To establish a PUR-degrading enrichment culture, the PUR plastisphere was used as an inoculum in a 20 mL glass vial containing 5 mL of ammonium mineral salts medium (AMS) containing 0.05% yeast extract, 0.01% CuSO_4_·5H_2_O, and a new piece of plasma-treated PUR foam as the sole carbon (C) source. The vial was incubated at 200 rpm and 25 ℃ until the enrichment culture was established. Then, a serial dilution of the enrichment culture in fresh PUR-containing AMS medium was performed. After 2 months, the fifth dilution was streaked onto AMS agar plates containing 0.6% Impranil, a water-borne PUR substrate (5), to isolate PUR-degrading strains. The obtained strain PHC1 was further confirmed for its ability to grow in the growth medium containing 0.6% Impranil as the sole C or N source (4). Here, we report the draft genome of strain PHC1.

The gDNA of the cell pellet of the liquid culture was extracted using FastDNA Spin Kit for Soil (MP Biomedical, Solon, OH). Library preparation and quality control were conducted using the NEBNext Ultra II DNA Library Preparation Kit (New England Biolabs, MA) and the KAPA Library Quantification Kit (Roche, Basel, Switzerland). Paired-end 2 × 150 bp sequencing was performed on the Illumina NovaSeq X platform (Illumina, CA). Raw reads were trimmed using Trimmomatic v0.4 with default parameters (6), and the genome was *de novo* assembled using SPAdes v4.0.0 with default parameters (7). Contigs shorter than 500 bp and those with a 77-mer coverage below 5 were excluded (https://github.com/ECBSU/oneliners). Genome statistics were obtained using QUAST v5.3.0 (8), while genome completeness and contamination were assessed with CheckM2 v1.0.2 (9). The primary bin corresponding to strain PHC1 exhibited 100% completeness and 0.21% contamination. Genome coverage was calculated using SAMtools v1.21 (10), following the alignment of the paired-end reads to the filtered contigs with Bowtie2 v2.5.4 with default parameters (11). The contigs were submitted to NCBI and annotated via the NCBI Prokaryotic Genome Annotation Pipeline v6.9 (12). Taxonomic assignment was performed using Average Nucleotide Identity (ANI) analysis using FastANI v.1.34 (13) comparing the draft genome to reference *Pseudomonas* genomes available in the NCBI database. The ANI result showed that the draft genome of strain PHC1 shares 93.99% identity with *Pseudomonas* fluorescens Pj0-1 (NCBI BioSample Accession number: SAMN02598267), a known low-density polyethylene degrader (14). The strain’s detailed information and annotation regarding PUR degradation is provided in Table 1.

**TABLE 1:** Genomic features of strains PHC1 and Genome-encoded candidate genes and pathways for PUR degradation in strain PHC1

Features			*Pseudomonas* sp. PHC1	
Assembly accession #			JBJXHJ010000000.1	
# of raw reads			16,698,692	
# of contigs			85	
Assembly length (bp)			6,181,688	
# of pair-end reads (trimmed and aligned)			15,229,613	
Genome coverage			727×	
Contig N_50_ (bp)			183,056	
Contig L_50_ (bp)			9	
GC content (%)			59.82	
Completeness (%)			100	
Contamination (%)			0.21	
# of coding sequences (CDS)			5,743	
# of CDS with proteins			5,563	
# of complete rRNA genes (5S, 16S, 23S)			1, 1, 2	
# of predicted tRNAs			55	

Gene/Cluster		Putative function	Protein accession number	Role in PUR degradation
Esterase		Hydrolysis of ester bonds	WP_410236556.1	Cleaves ester linkages, initiating PUR polymer breakdown (15)
Lipase		Breakdown of ester bonds	WP_410238278.1	Facilitates degradation of dispersed PUR particles (15)
			WP_410238357.1	
Serine hydrolase		Extracellular enzyme hydrolyzing ester bonds	WP_410237710.1	Inducible enzyme targeting PUR ester bonds (16)
			WP_410237772.1	
Urease	ureG	Urease accessory protein	WP_007950645.1	Contribute to urethane bond hydrolysis (17)
	ureF	Urease accessory protein	WP_236181945.1	
	ureE	Urease accessory protein	WP_007950631.1	
	ureC	Urease alpha subunit	WP_236181960.1	
	ureB	Urease beta subunit	WP_410236990.1	
	ureA	Urease gamma subunit	WP_039766748.1	
	ureD	Urease accessory protein	WP_236181963.1	

## Data Availability

Whole Genome Shotgun projects have been reported in GenBank under accession number JBJXHJ000000000.1. BioProject accession number is PRJNA1195118. BioSample accession number is SAMN45209548. Sequence Read Archive (SRA) accession number is SRR31683026.
